# Chiropractic care for paediatric and adolescent Attention-Deficit/Hyperactivity Disorder: A systematic review

**DOI:** 10.1186/1746-1340-18-13

**Published:** 2010-06-02

**Authors:** Fay Karpouzis, Rod Bonello, Henry Pollard

**Affiliations:** 1 Department of Chiropractic, Faculty of Science, Macquarie University, Sydney, NSW 2109, Australia; 2 Macquarie Injury Management Group, Macquarie University, Sydney, NSW 2109, Australia

## Abstract

**Background:**

Psychostimulants are first line of therapy for paediatric and adolescent AD/HD. The evidence suggests that up to 30% of those prescribed stimulant medications do not show clinically significant outcomes. In addition, many children and adolescents experience side-effects from these medications. As a result, parents are seeking alternate interventions for their children. Complementary and alternative medicine therapies for behavioural disorders such as AD/HD are increasing with as many as 68% of parents having sought help from alternative practitioners, including chiropractors.

**Objective:**

The review seeks to answer the question of whether chiropractic care can reduce symptoms of inattention, impulsivity and hyperactivity for paediatric and adolescent AD/HD.

**Methods:**

Electronic databases (Cochrane CENTRAL register of Controlled Trials, Cochrane Database of Systematic reviews, MEDLINE, PsycINFO, CINAHL, Scopus, ISI Web of Science, Index to Chiropractic Literature) were searched from inception until July 2009 for English language studies for chiropractic care and AD/HD. Inclusion and exclusion criteria were applied to select studies. All randomised controlled trials were evaluated using the Jadad score and a checklist developed from the CONSORT (Consolidated Standards of Reporting Trials) guidelines.

**Results:**

The search yielded 58 citations of which 22 were intervention studies. Of these, only three studies were identified for paediatric and adolescent AD/HD cohorts. The methodological quality was poor and none of the studies qualified using inclusion criteria.

**Conclusions:**

To date there is insufficient evidence to evaluate the efficacy of chiropractic care for paediatric and adolescent AD/HD. The claim that chiropractic care improves paediatric and adolescent AD/HD, is only supported by low levels of scientific evidence. In the interest of paediatric and adolescent health, if chiropractic care for AD/HD is to continue, more rigorous scientific research needs to be undertaken to examine the efficacy and effectiveness of chiropractic treatment. Adequately-sized RCTs using clinically relevant outcomes and standardised measures to examine the effectiveness of chiropractic care verses no-treatment/placebo control or standard care (pharmacological and psychosocial care) are needed to determine whether chiropractic care is an effective alternative intervention for paediatric and adolescent AD/HD.

## Background

Attention-Deficit/Hyperactivity Disorder (AD/HD) is considered to be one of the most frequently diagnosed disruptive behaviour disorders in childhood [1-5], with world wide prevalence rates of 8-12% [6]. The American prevalence rates range between 3-7% [1], and 4-12% [7]. The Australian prevalence rates show 11% of 6-17 year olds are diagnosed with this disorder [8], where as the English and Welsh AD/HD prevalence rates find 5% of 6-16 year olds have the disorder [9]. The *Diagnostic and Statistical Manual of Mental Disorders 4*^*th*^*Edition Text Revision *(DSM-IV-TR) [1], is the most widely used classification system for mental disorders [10,11]. The DSM-IV-TR characterises AD/HD as inappropriate, chronic levels of inattention, hyperactivity and impulsivity [1]. These children continually experience difficulties in academic achievement, and behavioural control, and as a consequence, they have difficulty in establishing positive relationships with family, authority figures and their peers [12-14]. As a result, much attention has been devoted to the development and evaluation of assessment and treatment for this disorder over the last fifty years [2,15-17]. The majority of the AD/HD literature is dedicated to the treatment of this disorder [2,15-18]. Most of this research can be found in the area of pharmacological therapies [12,16,17], with less emphasis in psychotherapy and other psychosocial interventions [19]. There is even less research in the area of AD/HD and complementary and alternative medicine (CAM) therapies [20,21].

Even though psychostimulants are the first line of therapy for paediatric AD/HD [2,12,22,23], the evidence reveals that up to 30% of these children do not show clinically significant outcomes, and others experience side-effects [12,24-28], and need to discontinue their medications [5,28]. For these children alternative strategies need to be considered and instigated [5,27,29].

In general, parents seek CAM therapies for their children for various reasons, such as they "feel mainstream medicine has let them down" [[30], p. 573], because a particular treatment was considered ineffective, fear of drug adverse effects and a need for more personal attention [31,32]. Furthermore, parents often prefer to try something 'natural' for their children [20,29,30,33].

It is obvious that parents with children diagnosed with AD/HD seek CAM therapies [20,34-38]. In fact, CAM therapies are sought more often by parents who have children with developmental and behavioural disorders such as AD/HD, than with any other condition [20,33,34,39]. Controversy over the safety and appropriateness of stimulant treatment has led to increased parental anxiety and the increased use of CAM therapies [20,31,40]. Major concern regarding the side effect profile of stimulant medications [29,31,34,41-43], has been the main reason parents have turned to alternative therapies [20,34-36,38,42,43]. Many parents and even teachers are receptive to, and have a preference for non-pharmacological or behavioural therapies for children with AD/HD [44,45]. In fact, parents and teachers show preferences for multidisciplinary approaches, which lead to reductions in medications [44,46,47].

In different surveys conducted around the world, CAM use for AD/HD ranged from 12% in Florida USA [37], 28% in Shaare Zedek, Israel [36], 54% in Boston USA [40], 64% in Perth Australia [38], and 68% in Melbourne Australia [34]. The American Academy of Paediatrics recognised the increasing use of CAM therapies in children and, as a result, assembled a Task Force on Complementary and Alternative Medicine in 2008 to address issues related to the use of CAM for this population [31]. This task force found that chiropractic care is one of the most common CAM practices provided at the professional level [31]. Other studies have also confirmed this finding [32,48,49]. Up to 10% of the US population seek care from chiropractors for non-musculoskeletal conditions [48,50,51]. Studies have confirmed that up to 14% of all chiropractic visits were for paediatric patients [39,52], and that chiropractors were the most common CAM providers visited by children and adolescents [31,52]. One study indicated that paediatric populations seek chiropractic care predominantly for non-musculoskeletal conditions or when asymptomatic [53].

A survey conducted in the USA on the presenting complaints of paediatric patients (under 18 years of age) for chiropractic care found that parents consulted chiropractors for their children's musculoskeletal (MSK) and non-musculoskeletal (non-MSK) conditions in addition to wellness care [53]. Of these paediatric chiropractic visits, 44% were for MSK conditions and 56% were for non-MSK conditions [53]. In this USA survey, included in the list of the most common non-MSK conditions parents sought chiropractic care for their children was hyperactivity [53].

A survey conducted in Australia of paediatric chiropractic care for children under 18 years of age found that parents (like their American counterparts) also sought care from chiropractors for their children's MSK and non-MSK complaints [54]. Within the Australian survey, parents consulted chiropractors for their children's non-MSK conditions, and included in that list were irritability, behavioural problems, AD/HD, and learning difficulties [54]. These two surveys have found that parents seek chiropractic care for their children's AD/HD, irritability, attentional and behavioural issues, as well as their learning disabilities from chiropractors, both in Australia, [54] and the USA [53].

Although figures appear low, parents are presenting to chiropractors with their children [50,53,55], looking for alternative therapies for AD/HD [42,43]. Anecdotally it has been suggested that AD/HD may be managed by chiropractic care, however to date no systematic review on the safety and efficacy of chiropractic care for paediatric and adolescent AD/HD has been conducted. A systematic review conducted to determine whether evidence exists for the therapeutic application of manipulation for paediatric health for musculoskeletal and non-musculoskeletal conditions revealed only low levels of scientific evidence [56]. In view of the large numbers of children and adolescents being diagnosed with AD/HD and the increased use of CAM therapies, of which chiropractic care is one of the most common, this review is relevant and important.

## Objective

To evaluate the evidence of the effect of chiropractic care for the treatment and/or management of children and adolescents with AD/HD.

## Methods

### Data Sources

The following electronic databases were searched by the primary author, with English language and human subjects as restrictions, from inception to July 2009: Index to Chiropractic Literature; Cochrane Central Register of Controlled Trials; Cochrane Library of Systematic Reviews; PubMed; MEDLINE-Ovid; PsycINFO; CINAHL (Cumulative Index to Nursing and Allied Health Literature); Mantis; Scopus and ISI Web of Science.

The following key words were used in the search strategy: "Attention Deficit Hyperactivity Disorder", "AD/HD", "Hyperactivity", "ADD", "Attention" and "chiropractic", "manipulative therapies", "spinal manipulation", "physical therapies", "complementary therapies", "alternate therapies".

### Searching other resources

The primary author conducted a hand search for articles held in the library at Macquarie University that did not have an e-copy available on-line. The chiropractic journals that were hand searched were DC Tracts (Vol. 4, 1992 - Vol. 14, 2002) and Journal of Manipulative and Physiological Therapeutics (Vol. 12, 1989). In addition, the reference lists of the retrieved papers were hand searched and screened to identify any additional studies that were not captured by electronic and manual searches. At the conclusion of these search procedures, all references were screened to avoid duplication.

### Study Selection

The primary author conducted the search and retrieved all relevant articles for the review and selected the articles that were Level I, II, III and IV evidence for chiropractic and AD/HD. All three reviewers agreed on the inclusion and the exclusion criteria outlined in Table 1. All three reviewers agreed on the Level of Evidence scale as outlined in Table 2. All full text articles retrieved were independently reviewed by at least two authors and the selection criteria were applied. Papers that did not meet the inclusion criteria were excluded from the systematic review.

**Table 1:** Inclusion and exclusion criteria used for the systematic review

Inclusion criteria	Exclusion criteria
Levels I, II and III evidence Chiropractic Intervention studies Study population: children age 0-17 years (inclusive) Diagnosis of AD/HD consistent with DSM-III, DSM-IV, DSM-IV-TR or ICD-10 criteria Diagnosis made by Paediatrician, Psychiatrist, Medical Doctor, Clinical or Educational Psychologist Validated Psychometric Outcome Measure as recommended by the American Academy of Child and Adolescent Psychiatry (AACAP 2007) (Table 3) Full-Text articles English language	Adults (18 yrs and over) Participant/s without a formal AD/HD diagnosis Qualitative studies Descriptive studies Observational studies Review/advice and/or opinion articles Articles that fall outside the NHMRC designated levels of evidence

**Table 2:** National Health and Medical Research Council (NHMRC) levels of evidence

Level	Intervention Studies
I	Systematic review of level II studies
II	Randomised controlled trial
III-I	Pseudo-randomised controlled trial (i.e. alternate allocation or some other method)
III-2	Comparative study with concurrent controls:
	•Non-random, experimental trial
	•Cohort study
	•Case-control study
	•Interrupted time series with a control group
III-3	Comparative study without concurrent controls:
	•Historical control study
	•Two or more single arm study
	•Interrupted time series without a parallel control group
IV	Case series with either post-test or pre-test/post-test outcomes

NB. Adapted from NHMRC Levels of evidence [58]

### Level of evidence

The scale of evidence adopted for this review was taken from the Cochrane Effective Practice and Organisation of Care (EPOC) Collaborative Review Group [57]. EPOC not only includes Level I and II evidence, but Level III evidence in its approach to systematic reviews.

The hierarchy of evidence is tabled below and adapted from the National Health and Medical Research Council (NHMRC) Levels of Evidence (Table 2) [58].

### Types of outcome measures

The primary outcomes considered in this review were the severity of symptoms of inattention, impulsivity, and hyperactivity. Outcome measures considered for inclusion were of ratings on standard, psychometrically sound, reliable and validated assessment questionnaires measuring changes in attentional, impulsive, and hyperactive symptoms over time. The outcome measures considered for inclusion are those used by the American Academy of Child and Adolescent Psychiatry [2]. These were chosen as they are considered common behaviour rating scales used in the assessment of AD/HD and for the monitoring of treatment. (Refer to Table 3)

**Table 3:** Common behaviour rating scales used in the assessment of AD/HD and monitoring of treatment.

Name of scale	Reference
Academic Performance Rating Scale (APRS)	The APRS is a 19-item scale for determining a child's academic productivity and accuracy in grades 1-6 that has 6 scale points; construct, concurrent, and discriminant validity data, as well as norms (n = 247), available (Barkley, 1990) [103]

AD/HD Rating Scale-IV	The AD/HD Rating Scale-IV is an 18-item scale using *DSM-IV *criteria (DuPaul et al., 1998) [104]

Brown ADD Rating Scales for Children, Adolescents and Adults	Psychological Corporation, San Antonio, TX http://www.drthomasebrown.com/assess_tools/index.html (Brown, 2001) [105]

Child Behaviour Checklist (CBCL)	Parent-completed CBCL and Teacher-Completed Teacher Report Form (TRF) http://www.aseba.org/index.html

Conners' Parent Rating Scale-Revised (CPRS-R)^a^	Parent, adolescent self-report versions available (Conners, 1997)[106]

Conners' Teacher Rating Scale-Revised (CTRS-R)^a^	(Conners, 1997) [106]

Conners' Wells Adolescent Self Report Scale	(Conners and Wells, 1997) [106]

Home Situations Questionnaire-Revised (HSQ-R), School Situations Questionnaire-Revised (SSQ-R)	The HSQ-R is a 14-item scale designed to assess specific problems with attention and concentration across a variety of home and public situations; it uses a 0-9 scale and has test-retest, internal consistency, construct validity, discriminant validity, concurrent validity, and norms (n = 581) available (Barkley, 1990)[103]

Inattention/Overactivity With Aggression (IOWA) Conners' Teacher Rating Scale	The IOWA Conners is a 10-item scale developed to separate the inattention and overactivity ratings from oppositional defiance (Loney and Milich, 1982) [107]

Swanson, Nolan, and Pelham (SNAP-IV) and SKAMP Internet site AD/HD.NET	The SNAP-IV (Swanson, 1992) [108] is a 26-item scale that contains *DSM-IV *criteria for AD/HD and screens for other *DSM *diagnoses; the SKAMP (Wigal et al., 1998)[109] is a 10-item scale that measures impairment of functioning at home and at school

Vanderbilt AD/HD Diagnostic Parent and Teacher Scales	Teachers rate 35 symptoms and 8 performance items measuring AD/HD symptoms and common comorbid conditions (Wolraich et al., 2003a) [110]. The parent version contains all 18 AD/HD symptoms with items assessing comorbid conditions and performance (Wolraich et al., 2003b) [111]

Note: AD/HD = attention-deficit/hyperactivity disorder.

^a ^The longer form should be used for initial assessment, whereas the shorter form is often used for assessing response to treatment, particularly when repeated administration is required.

Source: American Academy of Child and Adolescent Psychiatry [2]

### Types of interventions

More than 100 different techniques are used by the chiropractic profession [59]. Although chiropractic techniques have been evolving for 114 years a complete discussion of what constitutes a chiropractic technique is beyond the scope of this paper. Furthermore, chiropractic is not a unimodal approach for treatment and/or management of musculoskeletal and non-musculoskeletal conditions, and it is not synonymous with the term spinal manipulation [60]. Chiropractors are qualified providers of spinal manipulation, spinal adjustments and other manual treatments, exercise instruction and patient education [61], often encompassing the biopsychosocial principles of health [62]. For those that are interested in the variety of techniques used by chiropractors, a list from a survey conducted in Australasia and North America can be found in Additional file 1[63], and many others can be located on the web [64]. As a result, no chiropractic interventions were excluded from this review.

### Quality assessment

The methodological quality of the studies that qualified for inclusion was assessed using the five-point Jadad score [65] (Additional file 2), and a 15- item checklist which is not validated but was developed by Hawk and colleagues [50] from the CONSORT statement [66] (Refer to Additional file 3).

## Results

### Selection of Studies

The search strategy yielded 58 citations. Of these citations, 22 citations were of intervention studies, 12 from peer-reviewed journals [67-78], and 10 from non-peer-reviewed journals [79-88]. Two studies were excluded as full-text of these articles was not available [85,88] (Refer to Additional files 4 and 5).

Studies were then independently screened by the authors to decide whether the studies met the criteria for inclusion. The authors found that this screening process yielded no studies that were Level I or II evidence. Four Level III evidence studies were found, but they did not meet the inclusion criteria for this review (Refer to Table 4). Therefore, scoring studies for methodological quality was not necessary. The authors of this review were not blinded to the authors, institutions, or the journals of publication of the articles. Please see Additional files 4 and 5 for a table of all citations.

**Table 4:** Log of rejected trials

Citation	Inclusion Criteria Met	Inclusion Criteria Not Met
Goff et al 2000 [75]	Level III-3 evidence Uncontrolled, Non-random, experimental trial N = 41	Adult study population Criteria not stated for a diagnosis No formal diagnosis No validated psychometric measures used according to AACAP

Giesen et al 1989 [76]	Level III-3 evidence Uncontrolled, Non-random, Single-Subject Design Children 7-13 years N = 7 Diagnosed by Paediatrician Used a psychometric outcome measure	Criteria not stated for a diagnosis Did not use validated psychometric measures according to AACAP

Brzozowske and Walton 1980 [77]	Level III-3 evidence Uncontrolled, Non-random, experimental trial Children 9-17 years N = 13	No AD/HD diagnosis stated Did not use validated psychometric measures according to AACAP

Brzozowske and Walton 1977 [78]	Level III evidence Controlled Non-Random Clinical Trial Children 9-17 years N = 24 1 child diagnosed with Minimal Brain Damage (1950's and 1960's terminology for AD/HD) 1 child on Ritalin-implied AD/HD diagnosis	Criteria not stated for a diagnosis Most of the study population did not have a specified diagnosis Did not use validated psychometric measures according to AACAP

Note: AACAP: American Academy of Child and Adolescent Psychiatry

## Discussion

An important result of this review is that the authors found that no studies met the inclusion criteria for this topic. The natural conclusion one draws from such a discovery, is that no evidence of studies for or against this treatment (chiropractic care) for this condition (paediatric and adolescent AD/HD) using RCTs (Level II evidence) were found. The reviewers then questioned whether or not their eligibility criteria were too strict or inappropriately defined [89]. In fact, evidence at lower levels of the hierarchy of evidence, such as non-randomised, quasi-experimental group designs or single-subject experimental designs could exist and could contribute valuable information [90]. The reviewers discovered that no RCTs existed on the subject matter and after discussion and reviewing the EPOC guidelines the eligibility criteria were extended to include Level III evidence (Table 2). Despite this extension of evidence to include Level III evidence the four intervention studies that were found did not meet the inclusion criteria (Refer to Table 4).

Researchers have used the term 'empty' review when a search to address a research question yields no eligible studies [89,90]. At first this may appear as though the review has no intrinsic value. However, knowing that there are no studies of a particular type on a specific topic has the potential to generate meaningful and useful information [90]. For researchers, empty reviews serve the purpose of highlighting research gaps and directing future original research projects, as was the case for these authors. There was a gap in the knowledge that needed an answer to an important clinical question: "does chiropractic care have a role to play in the treatment and/or management of paediatric and adolescent AD/HD?"

The inclusion of a log of rejected trials is an important aspect of any systematic review [90]. As part of the Cochrane review process a log of rejected trials is expected, outlining the studies that were excluded as well as listing the reasons for their exclusion [91]. Table 4 outlines the rejected studies and the reasons they were rejected.

This 'empty review' allows for the opportunity to learn from the excluded studies. For instance: What were the predominant types of research designs used? What types of populations have been studied? Which types of chiropractic interventions have been tested? What types of outcome measures if any, were used?

According to this systematic review, 15 case studies have been published [67-69,71-74,79,82-88]; three case series [70,80,81]; one single subject design study (n = 7) [76]; two uncontrolled, non-random experimental trials (n = 41 and n = 13) [75,77]; and one controlled, non-random, experimental clinical trial (n = 24) [78] for AD/HD and chiropractic care. Of these, two studies targeted adult AD/HD populations [70,75], three studies targeted paediatric and adolescent populations [76-78]. It is obvious from this review that there is a paucity of studies on paediatric and adolescent AD/HD and that the most predominant type of research design is the case study.

As for the types of chiropractic interventions investigated it was not a homogeneous finding. The chiropractic profession has over one hundred different techniques [59], and there was no shortage of variety in the studies found for this review. The following were some of the techniques investigated in the chiropractic and AD/HD literature: Diversified, Gonstead, Sacro-Occipital Technique (SOT), Craniosacral Therapy, Pettibon, Toggle Recoil Technique, Thompson Technique, Torque Release Technique, Network Spinal Analysis, Chiropractic Biophysics, and Activator Technique. As part of the interventions described in the published articles, advice on exercise and/or dietary modifications was also given in conjunction with some form of chiropractic treatment in seven of the studies reviewed [67,69-71,79,81,86] (Refer to Additional files 4 and 5).

In regard to the outcome measures used in these studies very few chiropractors actually used validated psychometric measures, in fact only one paediatric study used a known psychometric measure i.e. Werry-Weiss-Peters Parent Rating Scales [76]. However, according to Miller and colleagues this psychometric measure is best used when AD/HD is present with mental retardation [92]. This study also used electrodermal activity of skin conductance, and cervical x-rays [76]. The only other studies that used a psychometric outcome measures were the two adult AD/HD studies. One study used the Test of Variables of Attention (TOVA) [70] and the other used the Conners' Continuous Performance Test (CCPT) [75]. When reviewing the literature it is important to evaluate whether the patients (i.e. children and adolescents) presented to a chiropractor for treatment of traditional musculoskeletal conditions or whether they presented with a primary diagnosis of AD/HD. In every single case study the parents presented their child or adolescent to the chiropractor with a primary complaint of AD/HD, and chose to seek chiropractic care for their child's or adolescent's AD/HD symptoms. An interesting finding was that chiropractors used outcome measures that they would traditionally use for musculoskeletal conditions (i.e. x-rays, thermal scans, and surface electromyography) for AD/HD. These types of outcome measures are not used for AD/HD symptomatology in AD/HD studies published in the medical literature. One study used thermal scans with surface electromyography (sEMG) pre and post intervention as a measure of outcomes [68]. Two studies used sEMG as outcome measures [69,70], and another two studies used paraspinal thermal scans [67,79]. Two studies used rating scales designed by the chiropractor rather than using established reliable and validated psychometric rating scales [69,78]. Furthermore, all of the studies used subjective statements of a child's improvement taken from parents and/or teachers, and even a bus driver [67]. In all fairness many case studies presented were retrospective (although many were ambiguous) in nature and as a result it is highly probable that these chiropractors did not have any intentions of publishing and as a result did not seek out and use appropriate outcome measures for AD/HD symptomatology. However, it must be noted that even those few studies that were prospective in nature the chiropractors involved did not seek and use appropriate outcome measures.

When conducting research in the area of AD/HD a good guide to use is the "Practice Parameters for the Assessment and Treatment of Children and Adolescents with Attention-Deficit/Hyperactivity Disorder" [2]. Choosing psychometric measures that are recommended by the American Academy of Child and Adolescent Psychiatry [2] (Refer to Table 3), ensures that the outcome measures have normative values and are likely to yield a measure of AD/HD behaviours that are reliable.

For clinicians, an empty review provides valuable information showing that there is no evidence in support of a treatment on the basis of the inclusion criteria used in the review process [89,90]. Furthermore, empty reviews inform decision makers in health care when there is lack of robust evidence in favour of (or against) a particular health care intervention [93]. As was found in this review, there is no robust evidence in favour of chiropractic care for paediatric and adolescent AD/HD. It is important that chiropractors seek out the best evidence available. However, the absence of RCTs in this area does not need to immobilize clinical decision making, nor does it necessarily justify the abandonment of an intervention [90]. According to Sackett and colleagues [94,95], clinical expertise can be defined as "the proficiency and judgment that individual clinicians acquire through clinical experience and clinical practice" [[94], p.71]. Responsible practitioners need to integrate this evidence with their clinical expertise and should apply a common sense approach to each individual patient. Furthermore, all health care providers have a responsibility to inform their patients when a particular intervention does not have scientific validation, and that all they have is clinical experience and anecdotal evidence to support their treatment strategy, which is in keeping within the scope of evidence based practices [96].

If the chiropractic profession chooses to conduct research in the area of paediatric and adolescent AD/HD then appropriate study designs need to be followed. The gold standard for claiming a particular intervention caused the desired effect is the randomised controlled clinical trial (RCT). The CONSORT group recommendations are suggested to develop a stringent a set of guidelines designed to improve the reporting of RCTs [97]. The CONSORT Group also developed an extension of the CONSORT Statement for non-pharmacologic treatments [98], which can be easily applied to chiropractic intervention studies. If these guidelines are used in the design of a RCT then a robust study can be designed to minimise the risk of bias (internal validity) and to account for the applicability of a trial's outcomes to the target population (i.e. generalisability or external validity) [99].

With the increase use of CAM therapies the CONSORT group have assessed the quality of randomised trials for paediatric CAM therapies. They found that only 40% of the CONSORT checklist items were included in the published articles [100]. In order for these types of studies to be a valid source of information about paediatric CAM therapies, they need to be conducted and reported with the highest possible standards [100]. Unfortunately, the searches for this systematic review did not uncover any RCTs for the use of chiropractic care in paediatric or adolescent AD/HD cohorts. Chiropractic researchers can learn from the CONSORT group in order to design, conduct and report trials that will be valid and applicable in the future.

Lastly, it is important for chiropractors and chiropractic researchers to report any risks, side-effects or adverse events in relation to chiropractic interventions. "Every healthcare intervention comes with risk, great or small, of harmful or adverse effects" [91]. In all the studies reviewed for this systematic review there was not one mention of side effects or adverse reactions except for one study in which one adolescent girl reported feeling 'high' after her first adjustment [81]. However, it can not be assumed that the determination of side-effects was a specific goals of any of the studies reviewed, as it was not explicitly stated. It is strongly recommended that future studies for these age groups should include side effect and adverse reaction data. According to the Cochrane review it is important to minimize bias when conducting reviews by including an evaluation of adverse effects [91]. However, to date only one narrative report [101], and one systematic review for paediatric spinal manipulation [102], have been conducted reporting adverse events. Despite these, there are not enough data to evaluate causation or incidence rates of these rare adverse events. The importance of a prospective population-based active surveillance study has been recommended [102], in order to assess the severity and frequency of adverse events as a result of chiropractic care within the paediatric population. It is recommended that clinicians who administer spinal manipulation to paediatric populations should inform the parents that spinal manipulations may cause rare but serious adverse events [102].

## Limitations

A limitation of this review is that the search strategy included a literature search of articles only in the English language. It is possible that other articles have been published on AD/HD and chiropractic care in non-English journals. Another limitation that needs to be considered is publication bias as unpublished literature and abstracts from conference proceedings were not sought. Furthermore, hand searches were only conducted for a limited number of chiropractic journals held in the Macquarie University library.

## Conclusions

The current finding for this systematic review has been classified as an 'empty review'. As a result, to date there is no high quality evidence to evaluate the efficacy of chiropractic care for paediatric and adolescent AD/HD. The claims made by chiropractors that chiropractic care improves AD/HD symptomatology for young people is only supported by low levels of scientific evidence. In the interest of paediatric and adolescent health, if chiropractic care is to continue for this clinical population, more rigorous scientific research needs to be undertaken to examine the efficacy and effectiveness of chiropractic treatment for AD/HD. Adequately-sized RCTs using clinically relevant outcomes and standardised measures to examine the effectiveness of chiropractic care verses no-treatment/placebo control or standard care (pharmacological and psychosocial care) are needed to determine whether chiropractic care is an effective alternative intervention for paediatric and adolescent AD/HD.

## Abbreviations

AD/HD: Attention-Deficit/Hyperactivity Disorder; ADD: Attention Deficit Disorder; DSM-IV-TR: Diagnostic and Statistical Manual of Mental Disorders 4^th ^Edition Text Revision; DSM-IV: Diagnostic and Statistical Manual of Mental Disorders 4^th ^Edition; DSM-III: Diagnostic and Statistical Manual of Mental Disorders 3^rd ^Edition; ICD-10: International Classification of Diseases 10^th ^Revision; CAM: Complementary and Alternative Medicine; CINAHL: Cumulative Index to Nursing and Allied Health Literature; AACAP: American Academy of Child and Adolescent Psychiatry; EPOC: Cochrane Effective Practice and Organisation of Care Collaborative Review Group; NHMRC: National Health and Medical Research Council; CONSORT: Consolidated Standards of Reporting Trials; RCT: Randomised Controlled Trial; CCPT: Conners' Continuous Performance Test; sEMG: Surface Electromyography.

## Competing interests

The authors declare that they have no competing interests.

## Authors' contributions

FK, RB and HP conceived the research project. All authors contributed to the writing of the manuscript. All authors read and approved the final manuscript

## Supplementary Material

Additional file 1 **Survey list of top 15 techniques used by chiropractors in Australasia and North America**. This table summarises the top 15 techniques used by chiropractors in Australasia and North America according to a survey conducted by Walker et al [72]. Click here for file

Additional file 2 **Jadad Five-Point Scale used to score studies**. Sourced from an article published by Jadad et al on assessing the quality of randomised clinical trials [74]. Click here for file

Additional file 3 **Modified CONSORT checklist**. Modified CONSORT checklist sourced from an article published by Hawk et al [50] used for assessing the quality of randomised controlled trials. Click here for file

Additional file 4 **Summary of intervention studies for chiropractic care for AD/HD from peer-reviewed journals**. The summary presented in this table is of intervention studies of chiropractic care for AD/HD from full text publications from peer-reviewed journals used for the systematic review. Click here for file

Additional file 5 **Summary of intervention studies for chiropractic care for AD/HD from non-peer-reviewed journals**. The summary presented in this table is of intervention studies of chiropractic care for AD/HD from non-peer-reviewed journals used for the systematic review. Click here for file
