# Supplementary material for: Chiropractic care for paediatric and adolescent Attention-Deficit/Hyperactivity Disorder: A systematic review
Source: Chiropr Osteopat. 2010 Jun 2;18:13. doi: 10.1186/1746-1340-18-13 (PMC2891800; doi:10.1186/1746-1340-18-13)
Supplement: Additional file 1 — Survey list of top 15 techniques used by chiropractors in Australasia and North America. This table summarises the top 15 techniques used by chiropractors in Australasia and North America according to a survey conducted by Walker et al [72]. [file 1746-1340-18-13-S1.DOC]

**Additional File 1**

Survey list of top 15 techniques used by chiropractors in Australasia and North America

| **Australasia** | **North America** |
| --- | --- |
| 1. Activator Technique | 1. Diversified |
| 2. Diversified | 2. Activator Technique |
| 3. Gonstead Technique | 3. Gonstead Technique |
| 4. Sacro-Occipital Technique (SOT) | 4. Manipulation |
| 5. Manual Adjustment | 5. Thompson Technique |
| 6. Applied Kinesiology (AK) | 6. Cox |
| 7. Drop Piece | 7. Acupuncture |
| 8. Soft Tissue/Massage | 8. Pressure Point Technique |
| 9. Neuro Emotional Technique (NET) | 9. Sacro-Occipital Technique (SOT) |
| 10. Thompson Technique | 10. Physiotherapeutics |
| 11. Manipulation | 11. Chiropractic Biophysics (CBP) |
| 12. Chiropractic Biophysics (CBP) | 12. Neuro Emotional Technique (NET) |
| 13. Acupuncture | 13. Electrical stimulation |
| 14. Network Spinal Analysis | 14. Activator Release Technique (ART) |
| 15. Trigger Point Therapy | 15. Trigger Point Therapy |

Note: List sourced from article published by Walker et al [63]
