# Supplementary material for: Chiropractic care for paediatric and adolescent Attention-Deficit/Hyperactivity Disorder: A systematic review
Source: Chiropr Osteopat. 2010 Jun 2;18:13. doi: 10.1186/1746-1340-18-13 (PMC2891800; doi:10.1186/1746-1340-18-13)
Supplement: Additional file 2 — Jadad Five-Point Scale used to score studies. Sourced from an article published by Jadad et al on assessing the quality of randomised clinical trials [74]. [file 1746-1340-18-13-S2.DOC]

**Additional File 2**

Jadad Five-Point Scale used to score studies

|  | **Yes** | **No** |
| --- | --- | --- |
| Study was described as randomised. | 1 | 0 |
| Study was described as double-blinded. | 1 | 0 |
| Description of withdrawals and dropouts was provided. | 1 | 0 |
| Methods to generate the sequence of randomisation were described and were  appropriate. | 1 | 0 |
| Methods to generate the sequence of randomisation were described and were  inappropriate. | -1 | 0 |
| Methods of double blinding were described and were appropriate. | 1 | 0 |
| Methods of double blinding were described and were inappropriate. | -1 | 0 |

Scoring: 0–2 = low quality; 3–5 = high quality.

Note: Sourced from Jadad Five-Point Scale used to score studies [65]
