# Supplementary material for: Chiropractic care for paediatric and adolescent Attention-Deficit/Hyperactivity Disorder: A systematic review
Source: Chiropr Osteopat. 2010 Jun 2;18:13. doi: 10.1186/1746-1340-18-13 (PMC2891800; doi:10.1186/1746-1340-18-13)
Supplement: Additional file 3 — Modified CONSORT checklist. Modified CONSORT checklist sourced from an article published by Hawk et al [50] used for assessing the quality of randomised controlled trials. [file 1746-1340-18-13-S3.DOC]

**Additional File 3**

**Modified CONSORT checklist**

| Checklist | Yes | No |
| --- | --- | --- |
| Power calculation to determine sample size was reported. | 1 | 0 |
| Required sample size was attained. | 1 | 0 |
| Methods of blinding were described. | 1 | 0 |
| Success of blinding was assessed | 1 | 0 |
| Baseline characteristics of groups were described | 1 | 0 |
| Primary outcome measure was clearly stated. | 1 | 0 |
| Validity and reliability of primary outcome measure(s) were established | 1 | 0 |
| Adequate description of treatment or procedure was included | 1 | 0 |
| Therapeutic time was equivalent between groups. | 1 | 0 |
| Co-interventions were avoided or controlled for. | 1 | 0 |
| Possible biases in design were described and accounted for | 1 | 0 |
| Attrition was less than 25%. | 1 | 0 |
| Comparison of dropouts versus completers was made. | 1 | 0 |
| Statistical analysis was appropriate to compare outcomes between groups. | 1 | 0 |
| Incidence of adverse events was reported | 1 | 0 |

Scoring: 0–5 = low quality; 6–10 = medium quality; 11–15 = high quality.

Note: Modified CONSORT Checklist sourced from Hawk et al [50]
