# Supplementary material for: Chiropractic care for paediatric and adolescent Attention-Deficit/Hyperactivity Disorder: A systematic review
Source: Chiropr Osteopat. 2010 Jun 2;18:13. doi: 10.1186/1746-1340-18-13 (PMC2891800; doi:10.1186/1746-1340-18-13)
Supplement: Additional file 4 — Summary of intervention studies for chiropractic care for AD/HD from peer-reviewed journals. The summary presented in this table is of intervention studies of chiropractic care for AD/HD from full text publications from peer-reviewed journals used for the systematic review. [file 1746-1340-18-13-S4.DOC]

**Additional File 4**

**Summary of intervention studies for chiropractic care for AD/HD from peer-reviewed journals**

| **Citation** | **Study Type**  **&**  **Level of Evidence** | **Study Population**  **Age** | **Diagnosis**  **of**  **Participants** | **Interventions** | **Outcome Measures Used** | **Summary of Findings** |
| --- | --- | --- | --- | --- | --- | --- |
| Wittman  et al 2009 [67] | Case Study  Level IV | n = 1  6 yr male | Medically diagnosed & medicated | Chiropractic adjustments, craniosacral therapy, movement exercise, nutritional support | Thermal Scans | Teacher and bus driver noted improvements.  Discontinuation of medication |
| Cassista  2009 [68] | Case Study  Level IV | n = 1  4 yr male | Paediatrician | Pettibon, Toggle Recoil, Thompson technique | Thermal Scans and surface electromyography (sEMG) | Mother reported improved behaviour, sleep, and appetite, less hyperactive, more focused at school |
| Bedell  2008 [69] | Case Study  Level IV | n = 1  7 yr female | Teacher | Torque Release  Technique, craniosacral therapy, dietary changes and nutritional supplements. | Surface electromyography (sEMG) and  Rating scale designed by chiropractor | Chiropractor noted improvements in behavioural assessments. Mother noticed overall change in attitude and in specific tasks. |
| Pauli  2007 [70] | Case Series  Level IV | n = 9  22-58 yrs old  (4 males, 5 females) | Not stated | Network Spinal Analysis | TOVA- Test of Variable of Attention, Surface electromyography (sEMG) | TOVA AD/HD score “significant change p=0.08”  sEMG “significant reduction p=0.08” |
| Young  2007 [71] | Case study  Level IV | n = 1  4 yr male | Educational psychologist, and General Practitioner | Activator for Atlas,  exercise and supplements | None Stated | Subjective feedback from parents, teachers, speech therapist child improved especially language skills |
| Lovett and Blum  2006 [72] | Case study  Level IV | n = 1  6 yr male | Not Stated | Cervical adjustments and Sacral Occipital Technique | None Stated | Parent and teacher reported child improved behaviour, writing and spelling. |
| Bastecki  et al 2004 [73] | Case study  Level IV | n = 1  5 yr male | Medically diagnosed and medicated | Chiropractic care-Clinical Biomechanics of Posture protocol, Diversified, drop table and Toggle techniques | None Stated | Medical doctor concluded reduction in symptoms so discontinued stimulant medication. |
| Elster  2003 [74] | Case study  Level IV | n = 1  9 yr male | Medically diagnosed and medicated | Upper cervical chiropractic care | None Stated | Doctor’s observation, patient and parent subjective report, and thermographic scans of spine.  All 6 conditions no longer present. Medications such as Depakote and Adderall discontinued.  Only half dose Wellbutrin (anti-depressant) |
| Goff et al 2000 [75] | Uncontrolled, non-random experimental trial  Level III-3 | n = 41  32 yr Adults | Not Stated | Chiropractic care | Conners’ Continuous Performance Test | Improved attention p<0.05 statistically significant results |
| Giesen et al 1989 [76] | Uncontrolled, non-random,  Single subject design  Level III-3 | n = 7  7-13 yrs old | Paediatrician | Chiropractic manipulative therapy | Werry-Weiss-Peters parent rating scales;  Electrodermal activity of Skin Conductance; Cervical x-rays; Motion Recorder scores | Werry-Weiss-Peters parent rating scales- 57% (4/7) show improvement in parental ratings of hyperactivity.  Electrodermal activity-57% (4/7) show improvement in level of autonomic activity of Skin Conductance Level;  Cervical x-rays- 57% (4/7) show improvement in chiropractic radiographic findings;  Motion Recorder scores-71% (5/7) showed a reduction in overt behaviour activity (mean actomotor scores)  “Results suggestive of improvement” |
| Brzozowske and Walton  1980 [77] | Uncontrolled, non-random experimental trial  Level III-3 | n =13  4 yr males  9-12 yrs;  9 Adolescents aged 13-17 yrs | MDs, Psychiatrist,  Clinical Psychologist,  (Diagnosis learning and behavioural impairments) | Chiropractic treatment | None Stated | “Neurological dysfunction and/or brain damage affecting behaviour and academic progress were effectively reduced or eliminated” |
| Brzozowske and Walton  1977 [78] | Controlled Non-Random Clinical Trial  Level III-2 | n = 24  Children and adolescents  6 children aged 5-12 yrs were in each group  6 adolescents in each group | Clinical Psychologist and Counsellor  One on Ritalin; 2 unspecified medication, 1 Minimal Brain Damage; 2 no Diagnosis specified. | Group A Chiropractic treatment (n=12)  Group B control group (medicated) (n=12) | 13 question impairment scale  designed by research team | Reduction in hyperactivity and improved attention span in both groups.  Medication group not as sustained Chiropractic Treatment group.  No statistics shown – although commented on -“statistically chiropractic treatment was 20-40% more effective than the commonly used medications” |

Legend: RCT: Randomised Controlled Trial; AD/HD: Attention Deficit Hyperactivity Disorder; N/A: Not Available
