# Supplementary material for: Chiropractic care for paediatric and adolescent Attention-Deficit/Hyperactivity Disorder: A systematic review
Source: Chiropr Osteopat. 2010 Jun 2;18:13. doi: 10.1186/1746-1340-18-13 (PMC2891800; doi:10.1186/1746-1340-18-13)
Supplement: Additional file 5 — Summary of intervention studies for chiropractic care for AD/HD from non-peer-reviewed journals. The summary presented in this table is of intervention studies of chiropractic care for AD/HD from non-peer-reviewed journals used for the systematic review. [file 1746-1340-18-13-S5.DOC]

**Additional File 5**

Summary of intervention studies for chiropractic care for AD/HD from non-peer-reviewed journals

| **Citation** | **Study Type** | **Study Population** | **Diagnosis**  **of**  **Participants** | **Interventions** | **Outcome Measures Used** | **Summary of Findings** |
| --- | --- | --- | --- | --- | --- | --- |
| Stone-McCoy &  Przybysz  2009 [79] | Case Study  Level IV | n = 1  3.5 yr male | Not stated | Toggle Atlas adjustments,  Diet,  Supplements, exercise | Paraspinal thermal scans | Parent and teacher reported decrease in hyperactivity |
| Pauli  2007 [80] | Case Studies  Level IV | Children | Medically diagnosed and medicated | Chiropractic adjustments and dietary advice | None Stated | Parent noted improvements.  Discontinuation of medications |
| Mileski & McClay  2003 [81] | Case Studies  Level IV | n = 1  9 yr male  13 yr male  17 yr female | Medically diagnosed and medicated | Chiropractic adjustments and Dietary advice | None Stated | Parent noted improvements.  Discontinuation of medications |
| Liesman  1998 [82] | Case Study  Level IV | Male | Medically diagnosed | Chiropractic adjustments | None Stated | N/A |
| Peet JB  1997 [83] | Case study  Level IV | n = 1  8 yr male | Medically diagnosed and medicated | Chiropractic Biophysics | X-Ray analysis | Parent and teacher reported decrease in hyperactivity, and increase attention and emotional control |
| Peet P  1997 [84] | Case study  Level IV | n = 1  8 yr male | Not Stated | Chiropractic Biophysics | None Stated | Parent reported decrease of symptoms in child. |
| Barnes  1995 [85] | Case study  Level IV | n = 1  11 yr male | Medically diagnosed | N/A | N/A | Parent reported improvement academically and attention span. |
| Barnes  1994 [86] | Case study  Level IV | n = 1  10 yr male | Not Stated | Chiropractic care,  Dietary advice and supplements | None Stated | Chiropractor stating child improving academically, behaviour fluctuating |
| Langley 1994 [87] | Case study  Level IV | n = 1  8 yr female | Medically diagnosed and medicated | Chiropractic adjustments of 1^st^ and 2^nd^ cervical vertebra | None Stated | Chiropractor reported bedwetting resolved, no longer needs special education classes, decrease in frequency and severity of seizures, decrease in medications by specialist. |
| Anderson and Partridge  1993 [88] | Case study  Level IV | N/A | N/A | N/A | N/A | N/A |

Legend: N/A: Not Available
